# LRP1B Suppresses Immunotherapy Efficacy in Lung Adenocarcinoma by Preventing Ferroptosis

**DOI:** 10.1002/cam4.70486

**Published:** 2024-12-11

**Authors:** Zi‐Hao Ke, Ying Chen, Tao Yu, Qi Zhang, Yan Xiang, Kai‐Hua Lu

**Affiliations:** ^1^ Department of Respiratory and Critical Care Medicine The First Affiliated Hospital of Ningbo University Ningbo Zhejiang China; ^2^ Department of Oncology The First Affiliated Hospital of Nanjing Medical University Nanjing Jiangsu China; ^3^ Medical Research Center The First Affiliated Hospital of Ningbo University Ningbo Zhejiang China; ^4^ Department of Oncology The Affiliated Taizhou People's Hospital of Nanjing Medical University Taizhou Jiangsu China

**Keywords:** biomarker, ferroptosis, immunotherapy, LRP1B, lung cancer

## Abstract

**Background:**

Immune biomarkers for non‐small‐cell lung cancer (NSCLC) are programmed death ligand 1 (PD‐L1) and tumor mutational burden (TMB). However, they cannot accurately predict the effectiveness of immunotherapy. Identifying appropriate biomarkers that can differentiate between beneficiary groups is imperative.

**Methods:**

We identified lipoprotein receptor–related protein 1B (LRP1B) mutation as a potential biomarker for immunotherapy by analyzing clinical data, combined with bioinformatics analysis. The effects of LRP1B on ferroptosis were assessed using qRT‐PCR, Western blotting, CCK‐8 assay, and flow cytometry. The potential mechanism underlying the regulation of ferroptosis by LRP1B was elucidated using qRT‐PCR, Western blotting, ChIP, and dual‐luciferase reporter gene assays.

**Results:**

Through the collection and analysis of clinical data, we had established that LRP1B mutations are closely associated with immunotherapy. Bioinformatics analysis revealed significant differences in the expression levels of PD‐L1 and TMB between patients with LRP1B mutation and wild‐type patients in lung adenocarcinoma (LUAD). Furthermore, we observed that patients with LRP1B mutation in LUAD had significantly higher levels of tumor‐infiltrating lymphocytes (TILs) than wild‐type patients. In addition, we found that patients with LRP1B mutation in LUAD had significantly prolonged progression‐free survival (PFS) compared to wild‐type patients. However, the differences of PD‐L1 expression, TILs, and PFS were not observed in patients with LRP1B mutation in lung squamous cell carcinoma (LUSC). These findings provided strong evidence that LRP1B mutation was a potential biomarker for immunotherapy in LUAD. Moreover, our in vivo experiments indicated that knockdown of LRP1B enhanced the efficacy of mPD‐1, and mechanistic studies revealed that LRP1B regulated the sensitivity of cells to ferroptosis by modulating the expression of SLC7A11 through altering the phosphorylation level of STAT3. Further analysis revealed that LRP1B knockdown promoted immunotherapy in vivo.

**Conclusions:**

Our results confirmed that LRP1B affected the efficacy of immunotherapy by modulating the sensitivity of NSCLC cells to ferroptosis. LRP1B mutations represent a highly promising immunotherapeutic biomarker for NSCLC.

## 1 Introduction

Lung cancer is a prevalent form of cancer that ranks among the top causes of cancer‐related deaths worldwide, posing a severe threat to human health and life [1]. The two major pathological subtypes of lung cancer are non‐small‐cell lung cancer (NSCLC) and small‐cell lung cancer (SCLC), with NSCLC further subdivided into lung adenocarcinoma (LUAD) and lung squamous cell carcinoma (LUSC) [2]. Advanced‐stage diagnosis is common in over 75% of NSCLC patients, and despite the availability of various treatment options, the 5‐year survival rate is still below 25% [3]. Immunotherapy has emerged as a revolutionary treatment method in recent years. PD‐1/PD‐L1 immune checkpoint inhibitors are a popular form of interventional immunotherapy for solid tumors. PD‐L1 has been validated as a recognized immunotherapy biomarker in multiple clinical trials [4]. Several studies have demonstrated that combining immunotherapy with chemotherapy can lead to improved therapeutic outcomes compared to chemotherapy alone, regardless of PD‐L1 expression [5]. Therefore, while focusing on PD‐L1, it is also essential to identify new biomarkers that can guide immunotherapy.

Performing next‐generation sequencing (NGS) on patients is a common method used to search for potential biomarkers that can guide therapeutic efficacy. Our analysis of NGS data from patients revealed lipoprotein receptor–related protein 1B (LRP1B) mutations that correlate with a favorable immune response. Low‐density LRP1B is located on chromosome 2 and has a gene sequence that spans over 500 kb in length, composed of 91 exons ranging from 77 to 1899 bases, encoding 4599 amino acids [6, 7]. LRP1B is known to be downregulated in multiple cancers [8, 9, 10, 11, 12, 13]. LRP1B was initially dubbed as lipoprotein receptor–related protein deletion due to its homozygous deletion being observed in 40% of NSCLC cell lines [7]. Subsequently, pure somatic deletions of LRP1B were also discovered in gastric cancer, breast cancer, glioma, esophageal squamous cell carcinoma, and oral squamous cell carcinoma [14, 15, 16, 17, 18], while promoter region methylation of LRP1B was found in thyroid cancer, gastric cancer, esophageal squamous cell carcinoma, and oral squamous cell carcinoma cell lines [13, 14, 17, 18]. Moreover, fusion between two exons was observed in glioma cell lines, resulting in premature termination codon appearance, and overexpression of miR‐548a‐5p and miR‐301b‐3p leading to the downregulation of LRP1B [13, 16, 19]. Chromosomal, epigenetic, and microRNA (miRNA)‐related mechanisms may jointly contribute to the low expression of LRP1B in tumors. Numerous studies have suggested that decreased expression of LRP1B contributes to tumor initiation and progression, while upregulation of LRP1B suppresses tumorigenesis [9, 11, 12, 13, 14, 18, 19, 20, 21]. Our clinical data revealed that approximately 15% of patients harbor LRP1B mutations, and these patients exhibit significant response to immunotherapy. Based on these findings, it is possible that LRP1B mutation could serve as a promising biomarker for immunotherapy. Therefore, further investigation into the correlation between LRP1B mutation and immunotherapy is warranted.

The impact of LRP1B gene mutation on immunotherapy has long been a topic of concern. In lung adenocarcinoma (LUAD), LRP1B is one of the 26 significantly mutated genes and ranks among the top five [22]. Scientists conducted a retrospective analysis of 101 patients with LRP1B mutations who underwent immunotherapy. The mutations were classified into three categories based on their potential pathogenicity: pathogenic (P), possibly pathogenic (LP), and variants of uncertain significance (VUS). A P change is defined as any genomic alteration that may result in a large number of deletions, truncations, or loss of function. If missense mutations are listed in the Catalog of Somatic Mutations in Cancer (COSMIC) database and have a FATHMM functional analysis score higher than 0.5 through hidden Markov models, they are further categorized as LP changes. VUS is defined as an error change score < 0.5 not included in COSMIC or FATHMM. The P/LP‐LRP1B mutant has a higher objective response rate (ORR) and higher tumor mutational burden (TMB) value in immunotherapy compared with the VUS‐LRP1B mutant [23]. Furthermore, studies on lung cancer, liver cancer, and melanoma have suggested that patients with LRP1B mutations tend to have higher TMB, which may confer greater benefits from immunotherapy [24, 25].

Ferroptosis is a distinct form of cell death that is iron‐dependent and nonapoptotic, separate from apoptosis, various forms of necrosis, and autophagy. RBMS1 has been found to promote the progression of lung cancer by inhibiting ferroptosis through upregulating the expression of SLC7A11 [26]. SOX2, a stem cell factor, has been shown to promote resistance to ferroptosis in lung cancer cells by upregulating SLC7A11, which can enhance the self‐renewal ability of these cells [27]. Moreover, certain drugs can promote ferroptotic cell death and inhibit tumors. Curcumin can induce ferroptosis of tumor cells by activating autophagy, thereby promoting the death of NSCLC [28]. Additionally, curcumin can trigger ferroptosis of lung cancer cells via the lncRNA H19/miR‐19b‐3p/FTH1 axis, thereby exhibiting antitumor effects [29]. Moreover, there is growing evidence indicating that ferroptosis plays a critical role in T cell–mediated immunotherapy. The expression of SLC7A11 in tumor cells can be inhibited by IFN‐γ released by CD8^+^T cells, resulting in enhanced ferroptosis and improved efficacy of immunotherapy [30]. TRYO3 activates the PI3K‐AKT‐NRF2 pathway, which upregulates the expression of SLC3A3, GPX4, FTH, and other genes. This reduces tumor cell sensitivity to ferroptosis, resulting in drug resistance during immunotherapy [31]. Our findings indicated a strong association between LRP1B and ferroptosis, and suggested that LRP1B had an impact on the effectiveness of immunotherapy through this mechanism.

## 2 Materials and Methods

### 2.1 The Collection of Patient Clinical Data

From May 19, 2017, to October 26, 2023, 61 patients with NSCLC who received PD‐1/PD‐L1 inhibitor therapy in the Department of Oncology of the First Affiliated Hospital of Nanjing Medical University and had NGS test data were collected. The therapeutic efficacy of patients was evaluated using the RECIST1.1 evaluation criteria. Clinical benefit of anti‐PD‐1 inhibitors was defined as durable clinical benefit (DCB: complete remission [CR], partial remission [PR], or stability > 6 months [SD > 6 months]) and no durable clinical benefit (NDB: progression within 6 months [PD] or < 6 months stable [SD ≤ 6 months]). The study was approved by the Ethics Review Committee of the First Affiliated Hospital of Nanjing Medical University. Written informed consent for the study has been waived.

### 2.2 Cell Lines and Culture Conditions

Four LUAD cell lines (A549, SPAC‐1, H1299, H1975) were purchased from Proceeds, Wuhan, China. A549, SPAC‐1, and H1299 cell lines were cultured in DMEM high‐glucose medium containing 10% fetal bovine serum and 1% penicillin and streptomycin. The H1975 cell line was grown in 1640 medium containing 10% fetal bovine serum and 1% penicillin and streptomycin. The cells were routinely cultured in a 37°C incubator with a concentration of 5% CO_2_.

### 2.3 RNA Isolation and Quantitative Real‐Time Polymerase Chain Reaction (qRT‐PCR)

Total cellular RNA was extracted using Animal RNA Drawer Kit (Beyotime, Shanghai, China) and reverse transcribed to cDNA using HiScript III RT SuperMix Kit (Vazyme, Nanjing, China). PCR was then performed using ChamQ SYBR qPCR Master Mix (Vazyme, Nanjing, China).

### 2.4 Construction of Overexpression Cell Lines

Overexpression cell lines were constructed using the CRISPR/Cas9 SAM system (Gene, Shanghai, China) with LRP1B sgRNA sequence CGAGCCATCGGAGGCATCAA. The target cells with good growth conditions were taken, and the target cells were plated 1 day before virus infection. On the day of infection, dCAS9‐VP64 lentiviral particles were added according to the experimentally designed groups to conduct the target cell infection experiment. Puromycin selection was initiated 3 days post‐infection. After the cells were stable after Puromycin screening, they were re‐infected with sgRNA‐MS2‐P65‐HSF1 lentivirus, screened with G418, and collected for cell detection or related functional studies.

### 2.5 Cell Transfection

LRP1B shNC, shLRP1B, SLC7A11 siNC, and siSLC7A11 were obtained from GenePharma (Shanghai, China). To overexpress SLC7A11, we cloned the full‐length gene into a pcDNA vector to generate a recombinant plasmid for SLC7A11 overexpression. The empty pcDNA vector served as a control. Transfection was carried out using Lipofectamine 3000 reagent (Invitrogen) for both RNA and plasmid.

### 2.6 Western Blot Assay

Cells were lysed by RIPA lysis buffer (Beyotime, Shanghai, China). Protein concentration was measured using BCA protein assay kit (Beyotime, Shanghai, China). Quantified protein samples were loaded onto sodium dodecyl sulfate polyacrylamide gel electrophoresis (SDS‐PAGE) gel and transferred to polyvinylidene fluoride (PVDF) membrane. PVDF membrane was blocked by QuickBloc blocking buffer (Beyotime, Shanghai, China). The membrane was then incubated at 4°C overnight with following antibodies: LRP1B (1:1000), SLC7A11 (1:1000), STAT3 (1:1000), p‐STAT3 (Tyr705) (1:1000), GAPDH (1:1000). Membranes were washed three times with TBST and then incubated with a horseradish peroxidase‐conjugated secondary antibody (Proteintech) for 1 h at room temperature, and visualized using a chemiluminescent substrate (Invitrogen, USA) and the Bio‐Rad Image Lab detection system (USA).

### 2.7 Luciferase Reporter Assay

The SLC7A11 promoter‐luciferase reporter system was constructed by ligating a DNA fragment encoding the human SLC7A11 promoter into Pezx‐FR03‐basic (FulenGen, Guangzhou, China). A dual‐luciferase reporter assay system (Promega) was used to measure the luciferase activities in cell lysates after cells were treated with Colivelin TFA. Renilla luciferase was used for the normalization of Luciferase values.

### 2.8 Chromatin Immunoprecipitation (ChIP) Assay

ChIP assays were performed using an EZ‐Magna ChIP kit (Millipore, Billerica, MA, USA). ChIP DNA was analyzed by qPCR with SYBR Green (Vazyme) on an ABI‐7500 (Applied Biosystems). The antibodies used are as follows: p‐STAT3 (Tyr705) (CST) and normal Rabbit IgG (Proteintech). The SLC7A11 promoter primers were used in this research: 1F, GAAGGTCTGTTCCGAATT, 1R, TGAGAAGCCTCCAGCAT; 2F, AGCATTGAGGTGGTGTC, 2R, TCGCCATTAGGAAAGTA; 3F, TGTTCCCTGAGATTCCT, 3R, GAGCAGCTCTTCTTTCC.

### 2.9 Glutathione (GSH) Assay

The GSH in cell lysates were assessed using GSH assay kits (Beyotime), respectively, according to the manufacturer's instructions.

### 2.10 Malondialdehyde (MDA) Assay

The MDA contents in cell lysates were assessed using MDA assay kits (Solarbio), respectively, according to the manufacturer's instructions.

### 2.11 Lipid ROS Assay

Lipid ROS level was analyzed by flow cytometry using C11‐BODIPY 581/591 (Invitrogen, USA). Cells were incubated with C11‐BODIPY 581/591 at a final concentration of 10 μM for 30 min at 37°C and washed three times with PBS. The mean fluorescence intensity (MFI) was determined by flow cytometry (CytoFLEX, Beckman Coulter, USA) with 488 nm excitation and 510 nm emission filters. At least 10,000 cells were analyzed. The results were analyzed by the CytExpert software (Beckman Coulter, USA). Three independent experiments were conducted.

### 2.12 Animal Studies

Six‐week‐old C57BL/6 mice were purchased from Vitalriver. Mouse LLC cells (1 × 10^6^ cells) in 200 μL of PBS were subcutaneously injected on the right flank of C57BL/6 mice. Seven days after inoculation, 100 μg mouse anti–PD‐1 antibody (BE0146, Bio X Cell) or PBS was injected intraperitoneally twice a week for a total of 5 injections. Tumor volume was measured using the formula: length × width^2^/2, where length is the longest diameter of the tumor and width is the shortest diameter. A mouse tumor volume of 1500 mm^3^ was set as the endpoint.

### 2.13 Statistical Analysis

Fisher's exact test or Chi‐squared test was used to compare clinical parameters and gene mutation status between DCB and NDB patients. The Kaplan–Meier method was used to analyze progression‐free survival (PFS). Comparisons between the two groups were conducted using the Student's *t*‐test, with each group analyzed in triplicate. SPSS v.23.0 and GraphPad Prism v.6 were used for analysis and visualization, and *p* < 0.05 were considered statistically significant (***p < 0.001; **p < 0.01; *p < 0.05).

## 3 Results

### 3.1 Characteristics of the Cohort of NSCLC Patients Receiving Immunotherapy

Table 1 presents clinical information for 61 patients, with 40 treated with DCBs and 21 with NDBs. Of these patients, 84% were male and 16% were female. LUAD accounted for 69% of the cases, with LUSC being the second most common histological subtype at 30%. First‐line immunotherapy was administered to 82% of the patients, whereas 18% received immunotherapy in second line or beyond. Table 2 shows that the effectiveness of immunotherapy was significantly correlated with tumor stage and therapy line (*p* < 0.05), but was independent of age, gender, smoking status, and histology (*p* > 0.05).

**TABLE 1:** Baseline of NSCLC patients.

Characteristics	*N* (%)
Age (years)	
< 65	13 (21%)
≥ 65	48 (79%)
Gender	
Male	51 (84%)
Female	10 (16%)
Smoking status	
Former/Current	39 (64%)
Never	22 (36%)
Histology	
Adenocarcinoma	42 (69%)
Squamous cell carcinoma	18 (30%)
Adenosquamous carcinoma	1 (1%)
Stage	
III	12 (20%)
IV	49 (80%)
Therapy line	
1st	50 (82%)
≥ 2nd	11 (18%)
Treatment plan	
ICIs + Chemotherapy	38 (62%)
ICIs + Anti‐angiogenic	4 (7%)
ICIs + Anti‐angiogenic + Chemotherapy	17 (28%)
Others	2 (3%)

**TABLE 2:** The relationship between immunotherapy efficacy and patient baseline characteristics.

Factor	DCB	NDB	*p*
Age (years)			
< 65	8	5	0.73
≥ 65	32	16	
Gender			
Male	35	16	0.26
Female	5	5	
Smoking status			
Former/Current	27	12	0.42
Never	13	9	
Histology			
Adenocarcinoma	28	14	0.38
Squamous cell carcinoma	12	6	
Adenosquamous carcinoma	0	1	
Stage			
III	11	1	0.03
IV	29	20	
Therapy line			
1st	36	14	0.02
≥ 2nd	4	7	

### 3.2 LRP1B Mutations Are Associated With Clinical Benefit From Immunotherapy

**FIGURE 1:**
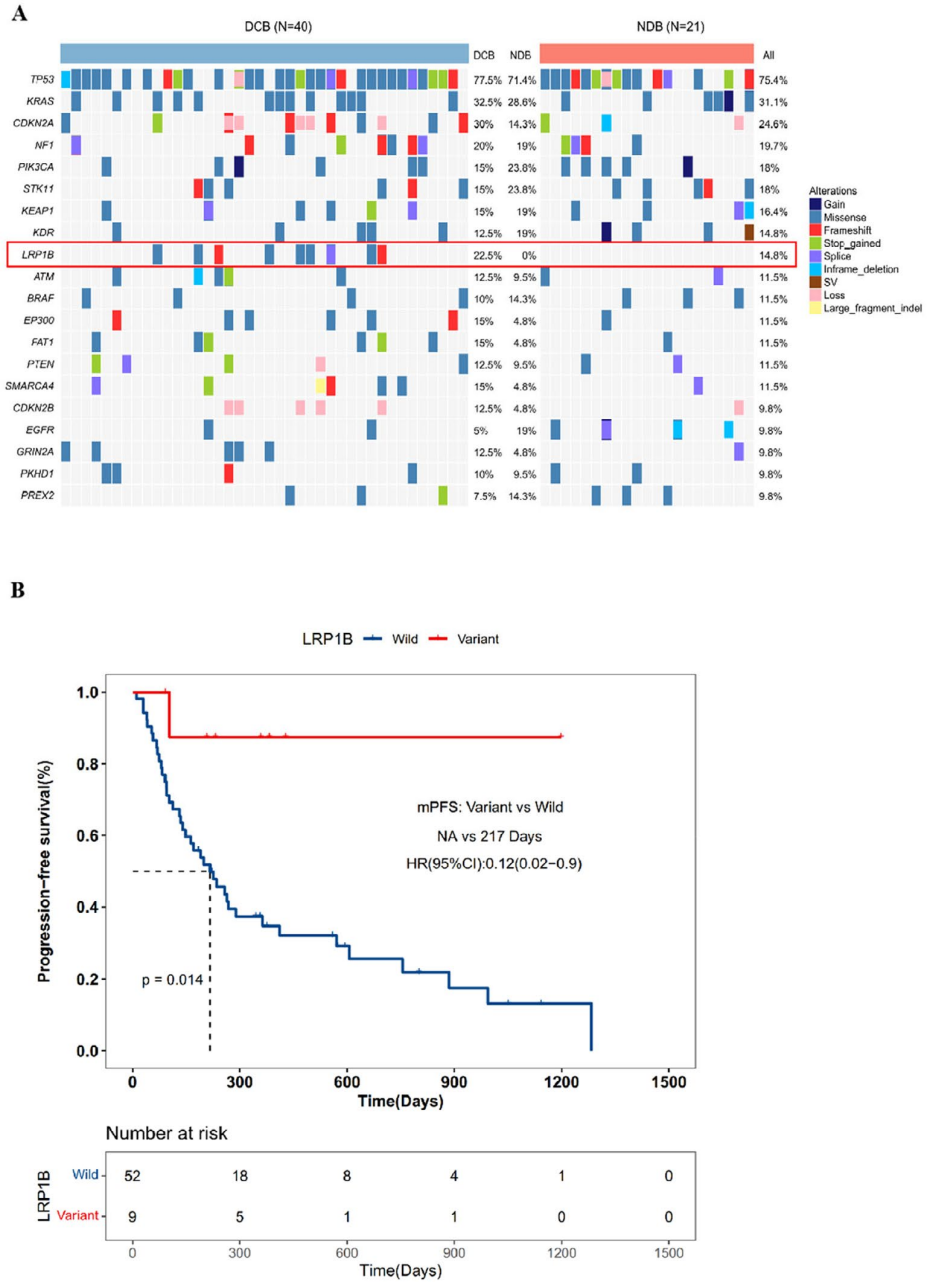
Overview of molecular characteristics linked to immunotherapy response. (A) The NGS results denote the mutated genes, which are listed below. Distinct mutations with high frequency were observed in DCB (left) and NDB (right) patients. (B) PFS Kaplan–Meier curves comparing LRP1B mutant and wild‐type patients.

To study how specific gene mutations relate to immunotherapy response, we initially concentrated on overall gene mutations. Figure 1A displays gene mutations in all patients, where TP53 mutations were found in over half (75.4%), while LRP1B and KDR mutations were present in 14.8% each, along with other common mutations such as KRAS (31.1%), STK11 (18%), and FAT1 (11.5%). The percentage of related gene mutations was consistent with previous reports [32]. We observed differences in gene mutations between DCB and NDB patients and identified distinct genes. Of these, LRP1B mutation (*p* = 0.01855) was significantly enriched in the DCB groups, respectively. We also investigated the correlation between LRP1B mutation and PFS. We noted that compared to patients with wild‐type LRP1B, patients with LRP1B mutations exhibited a significant prolongation in PFS, and this difference was statistically significant (Figure 1B). Prior research has indicated that LRP1B mutation may have a predictive function in immunotherapy [25]. As LRP1B mutation may be associated with immunotherapy benefit, we subsequently focused on it as our primary research target. Given the significant differences in gene mutation between LUAD and LUSC cell carcinoma, NSCLC was divided into these two subtypes and studied separately.

### 3.3 LRP1B Mutation Patients Have Higher PD‐L1 Expression Levels and TMB

**FIGURE 2:**
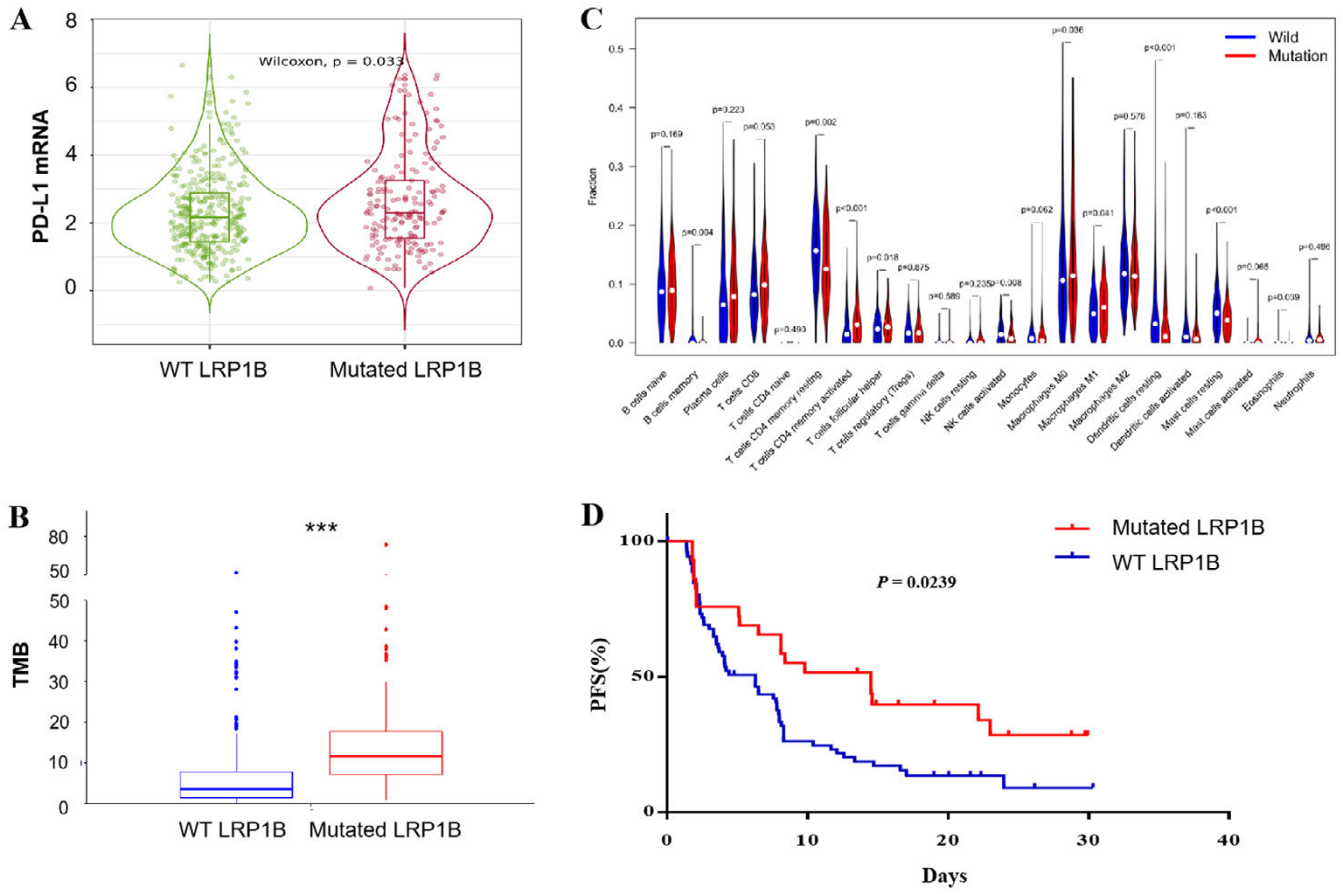
In LUAD, patients with LRP1B mutations have more positive factors for immunotherapy response. (A) The expression of CD274 (PD‐L1) LUAD by LRP1B mutation status. (B) TMB in LUAD stratified by LRP1B mutation status. (C) Relative abundance of tumor‐infiltrating leukocytes in LRP1B mutant vs. LRP1B wild‐type samples in LUAD and significantly different tumor‐infiltrating leukocytes. The data were presented as mean ± SD (***p < 0.001). (D) Kaplan–Meier survival analysis stratified by LRP1B mutation in LUAD (*n* = 103).

As PD‐L1 is currently the most widely used biomarker for immunotherapy, we sought to investigate the correlation between LRP1B mutations and PD‐L1. Our analysis of the TIMER 2.0 database revealed that PD‐L1 expression was notably higher in LRP1B‐mutant patients compared to those with LRP1B wild type in LUAD (Figure 2A). Meanwhile, in LUSC, the expression level of LRP1B was found to be independent of PD‐L1 expression levels (Figure S1A). TMB is another crucial biomarker for immunotherapy. Through analysis of the TCGA database, we discovered that LRP1B‐mutated patients had significantly higher TMB than wild‐type patients in both LUAD and LUSC (Figure 2B, Figure S1B). The tumor immune microenvironment significantly impacts the success of immunotherapy. Our analysis of the TCGA database revealed that memory B cells, CD8^+^ T cells, activated CD4 memory T cells, and M1 macrophages were significantly higher in LRP1B‐mutated patients with LUAD than in wild‐type patients (Figure 2C, Table 3). Conversely, we observed no corresponding changes in LUSC (Figure S1C). We utilized the cBioPortal database to gather a dataset of NSCLC immunotherapy, classified based on LUAD and LUSC. Our findings demonstrated that in LUAD, LRP1B‐mutant patients exhibited improved PFS compared to wild‐type patients (Figure 2D). However, in the LUSC group, LRP1B‐mutant patients did not exhibit significant PFS benefit when compared to wild‐type patients (Figure S1D). In summary, we believed that LRP1B mutation was a promising biomarker for immunotherapy in LUAD.

**TABLE 3:** The differential expression of Figure 2C.

Cell	*p*
B cells memory	0.003667345
T cells CD4 memory resting	0.002386988
T cells CD4 memory activated	0.000785198
T cells follicular helper	0.01777341
NK cells activated	0.008156979
Macrophages M0	0.036320664
Macrophages M1	0.040670206
Dendritic cells resting	6.78E‐05
Mast cells resting	8.23E‐05
Eosinophils	0.039188428

### 3.4 The Relationship Between LRP1B and Ferroptosis

**FIGURE 3:**
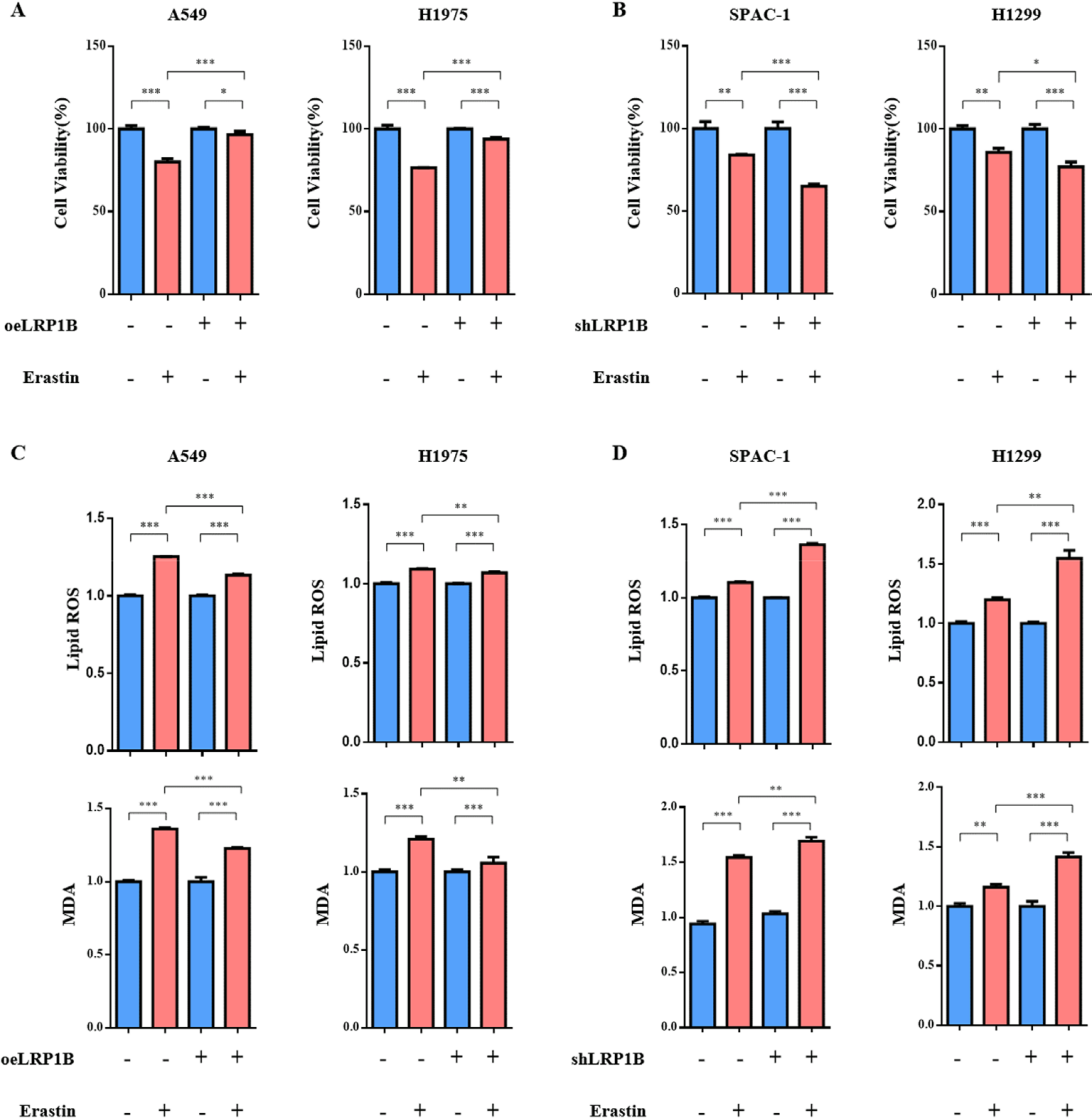
LRP1B inhibited erastin‐induced ferroptosis. Cells were treated with DMSO or 10 μM erastin for 24 h. (A) The CCK‐8 assay illustrated that overexpressing LRP1B further decreased the erastin‐induced reduction in cell survival rate for A549 and H1975 cells. (B) Reducing LRP1B expression further amplified the erastin‐induced decrease in cell survival rate for SPAC‐1 and H1299 cells. (C) LRP1B overexpression significantly reduced erastin‐induced increase in ROS and MDA levels. (D) LRP1B knockdown further increased MDA and ROS levels. The data were presented as mean ± SD (***p < 0.001; **p < 0.01; *p < 0.05).

To investigate the function of LRP1B in LUAD, we first measured LRP1B mRNA expression in A549, H1975, SPAC‐1, and H1299 cell lines (Figure S2A). Subsequently, we transfected LUAD cells with LRP1B overexpression plasmid and shRNA and confirmed the efficacy of LRP1B overexpression and knockdown using qRT‐PCR (Figure S2B) and western blotting (Figure S2C). Although previous research has focused mainly on the tumor suppressor function of LRP1B, its effect on immunotherapy remains unclear. Therefore, further investigation may be necessary to explore this phenomenon in greater detail. New research has indicated that the effectiveness of immunotherapy is linked to T cell–mediated ferroptosis [30]. We hypothesized that LRP1B could affect ferroptosis and thus treated LUAD cells with the ferroptosis inducer erastin to validate our hypothesis. Our findings showed that LRP1B overexpression reduced the impact of erastin‐induced cell death, as compared to the control group (Figure 3A), while LRP1B knockdown promoted erastin‐induced ferroptosis (Figure 3B). To observe the level of cell ferroptosis, we measured two important indicators, lipid ROS and MDA. Overexpression of LRP1B resulted in a significant reduction of elevated levels of lipid ROS and MDA caused by erastin, whereas knockdown of LRP1B promoted an increase in lipid ROS and MDA levels induced by erastin (Figure 3C,D). Collectively, our data showed that LRP1B played a critical role in protecting cells from ferroptosis.

### 3.5 The Regulatory Relationship Between LRP1B and SLC7A11

**FIGURE 4:**
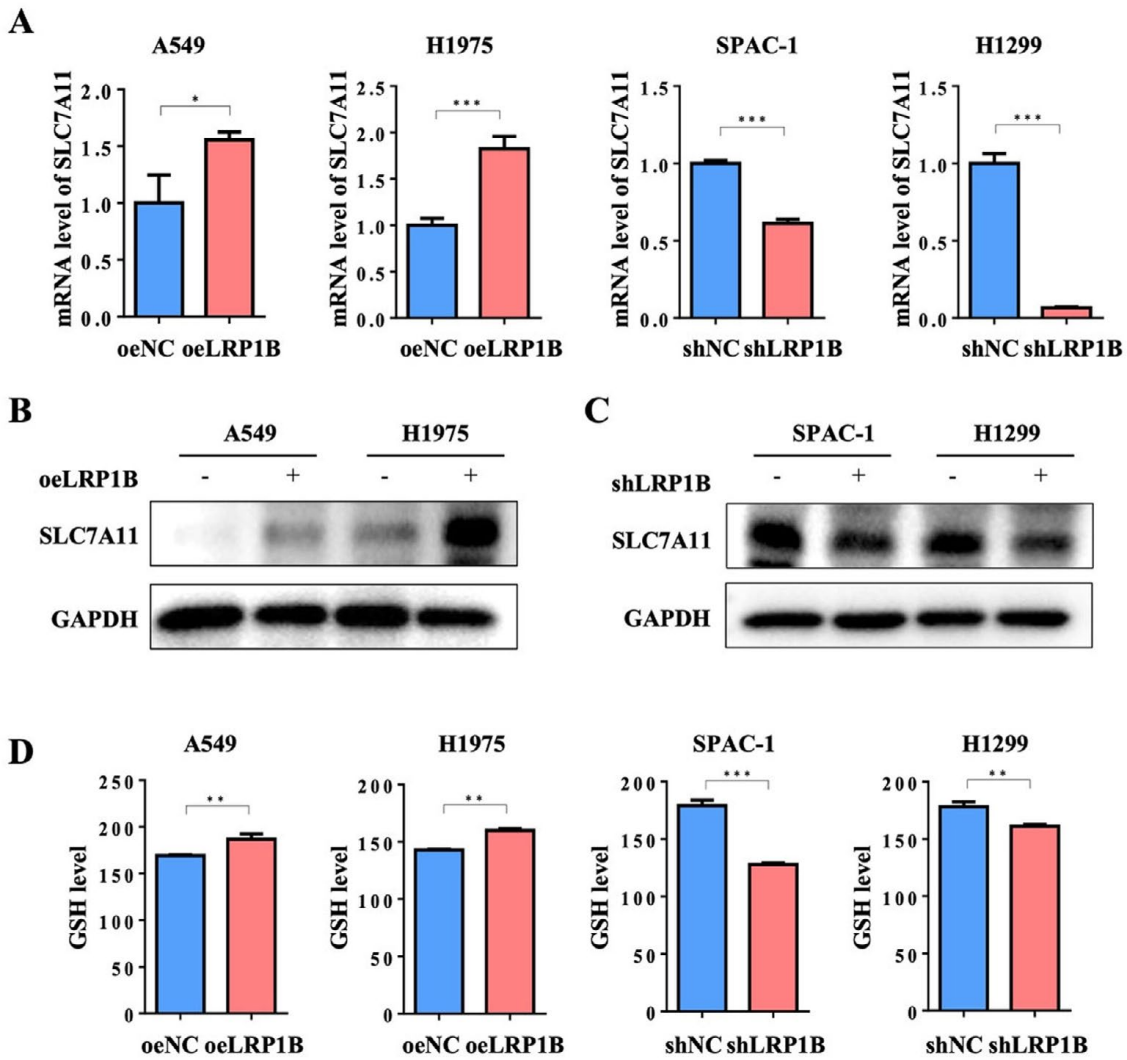
LRP1B could affect the expression of SLC7A11. (A) LRP1B could affect the mRNA expression level of SLC7A11. (B) LRP1B could affect the protein expression level of SLC7A11. (C) LRP1B could affect the level of intracellular GSH. The data were presented as mean ± SD (***p < 0.001; **p < 0.01; *p < 0.05).

Based on the TCGA database, we found a significant association between LRP1B and SLC7A11 (Table 4). SLC7A11 serves as a component of System Xc‐, which facilitates the transportation of cystine molecules into the cell, and cystine generates GSH through a series of metabolic reactions [33, 34, 35]. To assess the impact of LRP1B on SLC7A11 expression, we conducted qRT‐PCR and Western Blot analyses. Our results revealed that LRP1B overexpression led to elevated mRNA and protein levels of SLC7A11, while LRP1B knockdown resulted in reduced mRNA and protein levels of SLC7A11 (Figure 4A,B). These findings suggested that LRP1B played a role in regulating transcription and translation horizontally, thereby influencing the expression of SLC7A11. We further measured intracellular GSH levels and found that LRP1B overexpression increases the content of intracellular GSH, and decreases while LRP1B knockdown (Figure 4C).

**TABLE 4:** LRP1B‐related gene table.

Gene	Correlation coefficient	*p*
LRP1B	1	0
TRIM16L	0.345	7.77e‐12
PGD	0.339	1.81E‐11
SLC7A11	**0.326**	**1.77E‐10**
CYP4F11	0.322	2.75E‐10
ABCB6	0.321	2.93E‐10
CA8	0.32	2.7E‐10
BTBD11	0.319	3.3E‐10
PPP2R2C	0.318	3.08E‐10
SRXN1	0.315	4.81E‐10
OSGIN1	0.314	5.31E‐10

*Note:* The bold values presented in Table 4 indicates that among the genes with a strong correlation to LRP1B, we have noted the ferroptosis‐related gene SLC7A11.

### 3.6 LRP1B Inhibited Ferroptosis by Upregulating SLC7A11

**FIGURE 5:**
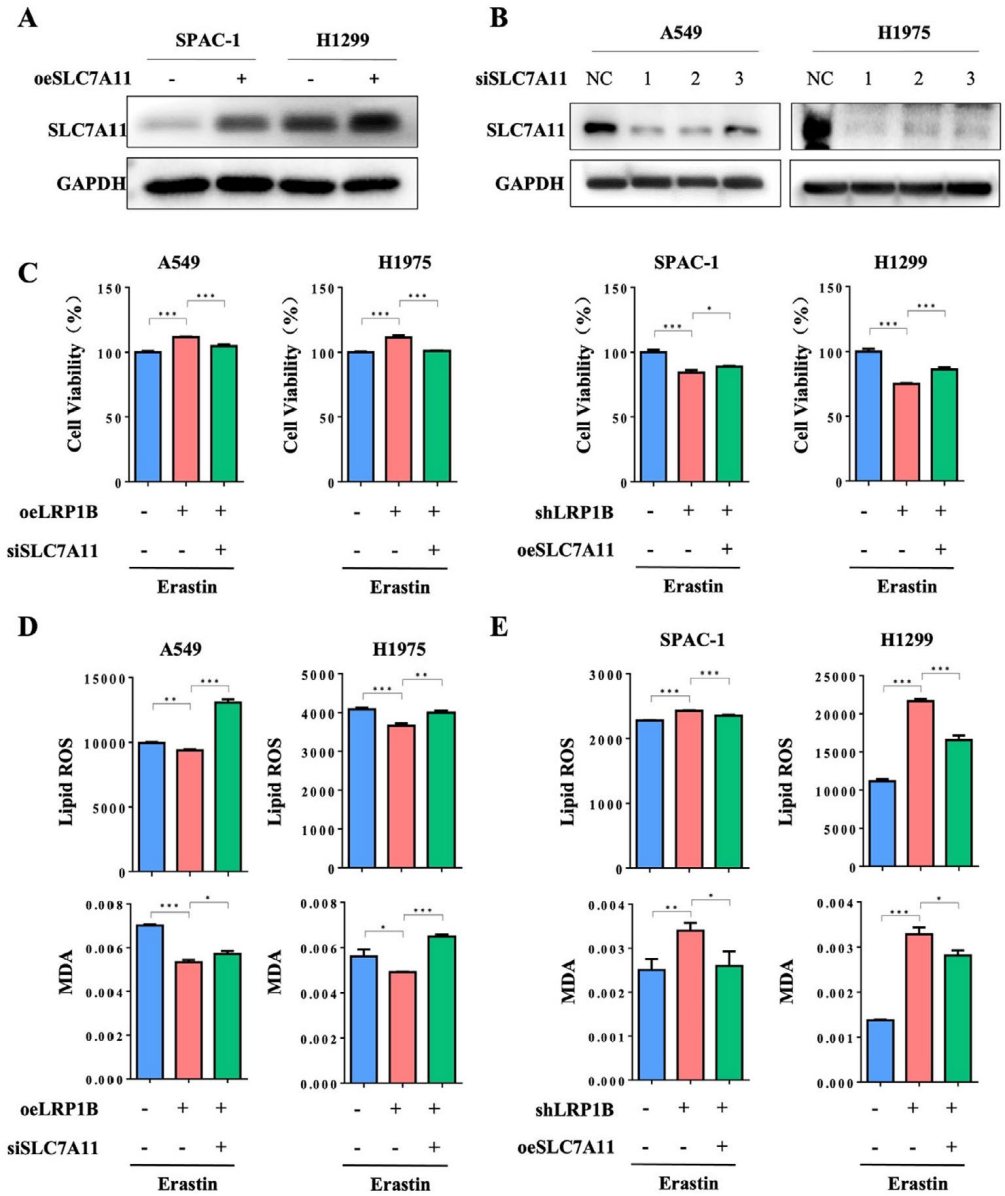
LRP1B regulates ferroptosis through SLC7A11. Cells were treated with DMSO or 10 μM erastin for 24 h. (A and B) The protein expression level of SLC7A11 after overexpression and knockdown of SLC7A11. (C) Knockdown of SLC7A11 significantly reduced the cell survival rate after erastin induction, while overexpression of SLC7A11 significantly increased the cell survival rate after erastin induction. (D) Knockdown of SLC7A11 significantly increased erastin‐induced Lipid ROS and MDA production. (E) Overexpression of SLC7A11 significantly reduced erastin‐induced Lipid ROS and MDA production. The data were presented as mean ± SD (***p < 0.001; **p < 0.01; *p < 0.05).

To investigate whether LRP1B affects ferroptosis through SLC7A11, we performed reversal experiments. We first confirmed the validity of the plasmids by overexpressing SLC7A11 in SPAC‐1 and H1299 cell lines, and knocking down SLC7A11 in A549 and H1975. The results are shown in Figure 5A,B. Next, CCK‐8 experiments confirmed that knockdown of SLC7A11 reduced cell survival after erastin induction, while overexpression of SLC7A11 improved cell survival after erastin induction (Figure 5C). Additionally, we found that knockdown of SLC7A11 in LRP1B‐overexpressing cell lines reversed the erastin‐induced reduction of intracellular lipid ROS and MDA levels caused by LRP1B overexpression (Figure 5D). On the other hand, overexpression of SLC7A11 in LRP1B‐knockdown cell lines could reverse the erastin‐induced increase in intracellular lipid ROS and MDA levels caused by LRP1B knockdown (Figure 5E). Overall, our data indicate that LRP1B can regulate ferroptosis through SLC7A11.

### 3.7 LRP1B Regulates SLC7A11 Through STAT3

**FIGURE 6:**
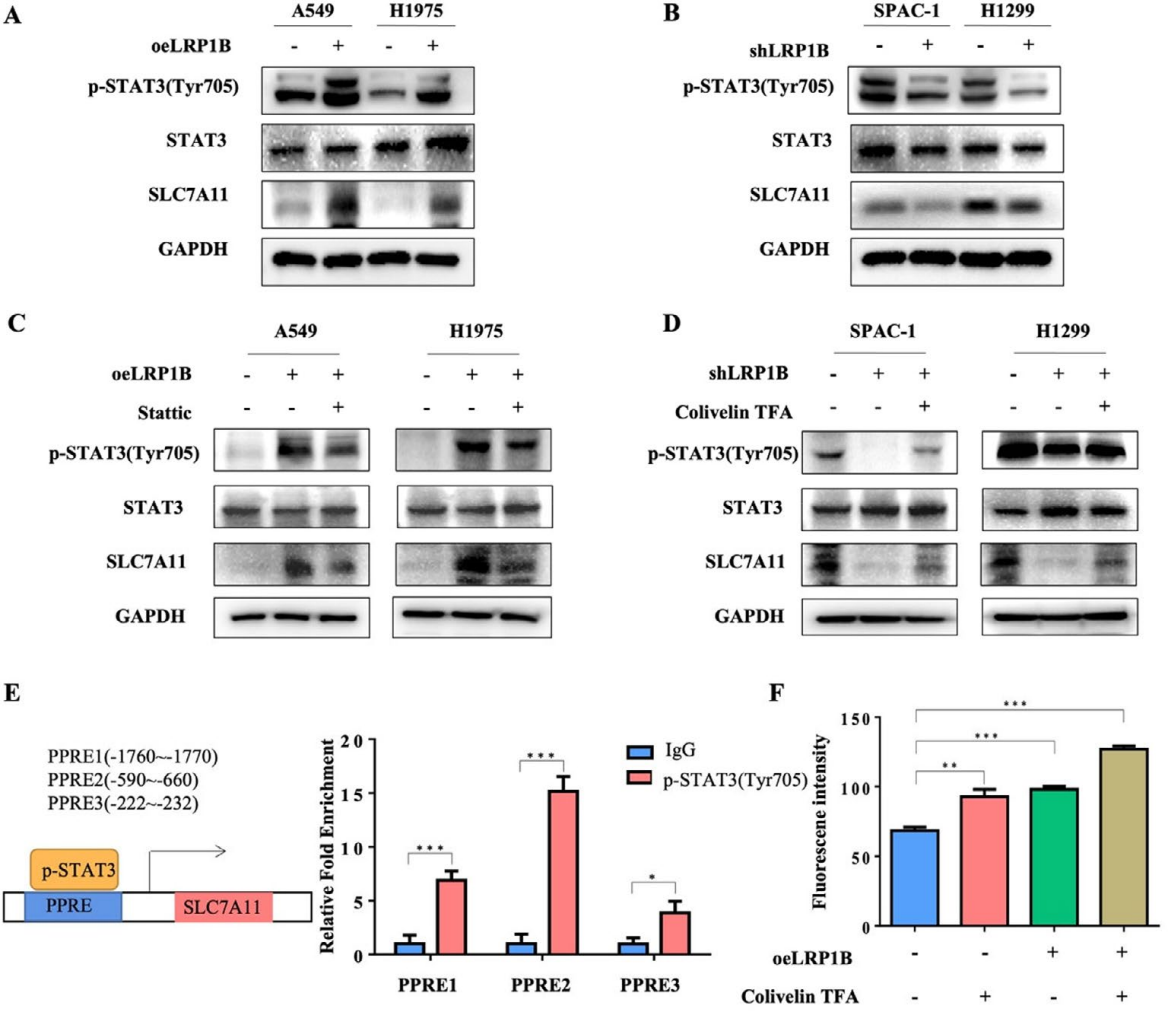
The activation of p‐STAT3 upregulated the expression of SLC7A11. Cells were treated with DMSO, 20 uM Stattic, 50 μg/mL Colivelin TFA, or 10 μM erastin for 24 h. (A) After overexpression of LRP1B, STAT3 phosphorylation level increased. (B) After knockdown of LRP1B, the phosphorylation level of STAT3 decreased. (C) STAT3 phosphorylation inhibitor Static can reverse the increase of SLC7A11 protein level caused by overexpression of LRP1B. (D) The STAT3 phosphorylation agonist Colivelin TFA can reverse the decrease of SLC7A11 protein level caused by knockdown of LRP1B. (E) CHIP experiment showed that p‐STAT3 could bind to SLC7A11 promoter region. (F) Luciferase reporter assay showed that the increase of STAT3 phosphorylation level could enhance the transcription activity of SLC7A11 promoter. The data were presented as mean ± SD (***p < 0.001; **p < 0.01; *p < 0.05).

Studies have shown that overexpression of LRP1B can promote the phosphorylation of STAT3 [21]. To explore the relationship between LRP1B, STAT3, and SLC7A11, we first verified this with Western blot. Our findings revealed that the expression levels of p‐STAT3 and SLC7A11 were elevated upon LRP1B overexpression (Figure 6A). Conversely, knockdown of LRP1B decreased STAT3 phosphorylation and SLC7A11 expression (Figure 6B). Therefore, we hypothesized that LRP1B affects the transcription of SLC7A11 by regulating the phosphorylation level of STAT3. To test this hypothesis, we treated A549‐oeLRP1B and H1975‐oeLRP1B cells with the STAT3 phosphorylation inhibitor Stattic. Western blot results showed that the expression of SLC7A11 decreased after Stattic treatment (Figure 6C). SLC7A11 expression was elevated by shLRP1B using the STAT3 phosphorylation agonist Colivelin TFA (Figure 6D). Upon phosphorylation, STAT3 acts as a transcription factor by binding to the promoter region of genes within the nucleus and thereby stimulating the transcription of downstream genes. Our hypothesis was that p‐STAT3 has the ability to bind to the promoter region of SLC7A11 and stimulate its transcription. As expected, a CHIP experiment showed that p‐STAT3 could bind to the SLC7A11 promoter (Figure 6E). Furthermore, a Luciferase reporter assay showed that p‐STAT3 could promote the transcription of SLC7A11 (Figure 6F). In summary, LRP1B can promote the phosphorylation of STAT3 and further promote the expression of SLC7A11.

### 3.8 Knockdown of LRP1B Facilitates Immunotherapy In Vivo

**FIGURE 7:**
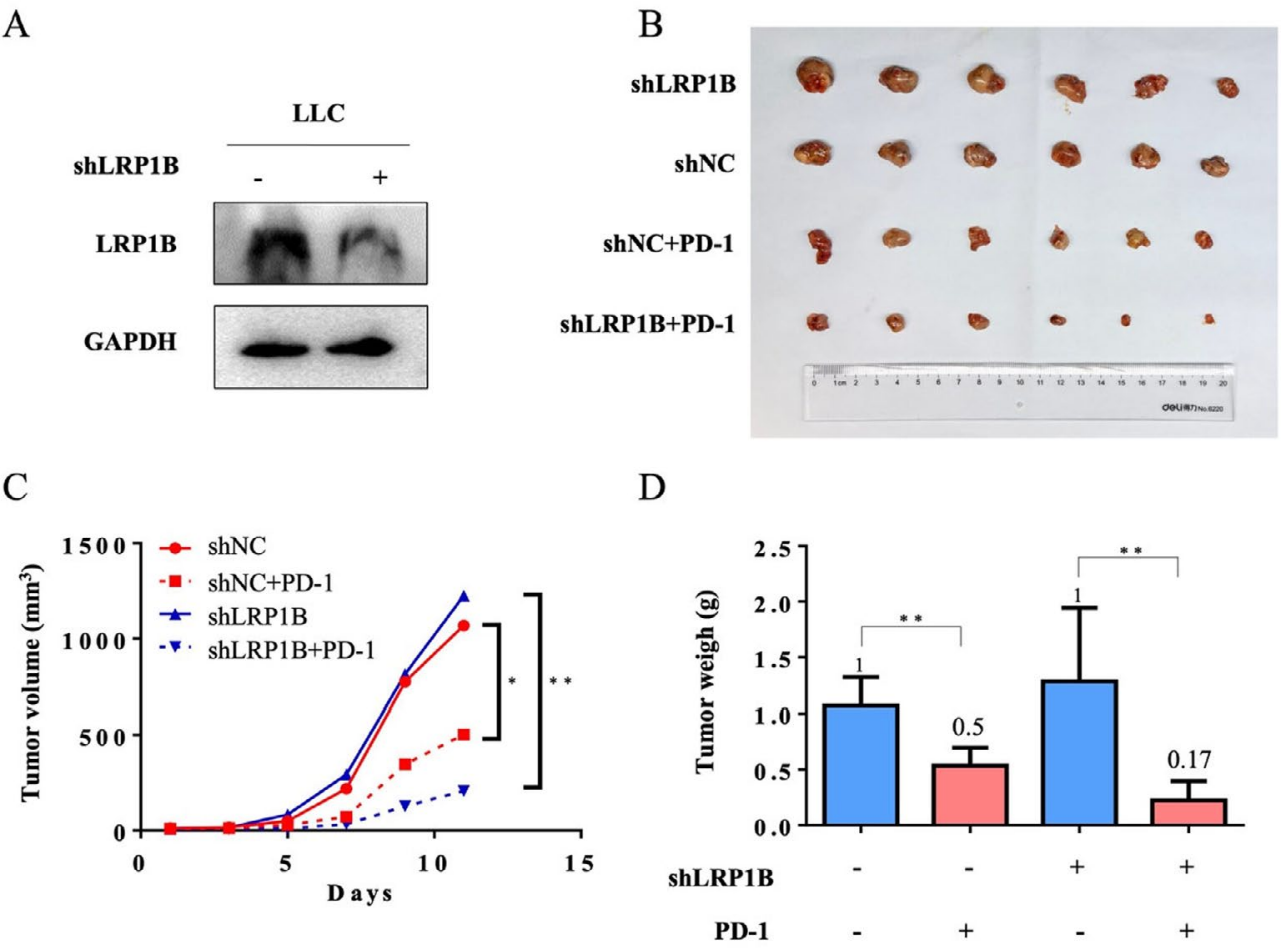
Decreased LRP1B expression confers immunotherapy. (A) Validation of LRP1B protein knockdown in LLC cells after transfection with LRP1B shRNA. (B) Tumor size, (C) tumor volume, (D) tumor weight from mPD‐1‐treated or untreated group after injection of shNC/shLRP1B LLC cells. The data were presented as mean ± SD ( **p < 0.01; *p < 0.05).

To further examine LRP1B's potential impact on immunotherapy, we created a cell line derived from mice with knockdown of LRP1B expression (Figure 7A). Afterward, we established a subcutaneous tumor model in C57BL/6 mice by subcutaneously injecting LLC‐NC and LLC‐shLRP1B cells. Seven days after injection, we intraperitoneally injected mouse PD‐1 antibody and monitored tumor growth until it reached the expected maximum volume, after which the tumor was harvested. Our findings indicated that the size and weight of tumors were significantly smaller in the group with LRP1B knockdown compared to the control group (Figure 7B,C). These findings suggest that LRP1B knockout enhances the efficacy of immunotherapy.

## 4 Discussion

Immunotherapy has brought fresh treatment options for patients suffering from late‐stage lung cancer. Despite its excellent curative effect, studies have shown that only about 20% of patients benefit from it [36]. Additionally, immunotherapy is an expensive treatment option and can cause side effects. Thus, it is crucial to pinpoint suitable biomarkers for identifying individuals who could profit from the treatment. Currently, PD‐L1 and TMB are the most extensively used biomarkers in clinical settings with abundant evidence‐based medicine. However, their hierarchical screening of patients is still limited, and there is a need to screen for new biomarkers to accurately apply immunotherapy in clinical practice.

NGS is essential in the treatment of lung cancer. The patient gene information provided by NGS provides an important basis for clinical decision‐making. Compared to traditional detection methods, NGS has the capability to recognize gene abnormalities that were previously unknown. In this study, we identified that LRP1B mutation is a potential biomarker for immunotherapy.

Previous studies on LRP1B have mainly focused on its antitumor function. However, the impact of LRP1B on immunotherapy and its mechanism are still unclear. First, we analyzed the relationship between the mutation status of LRP1B and CD274, TME, TMB, and PFS in patients who received immunotherapy from public databases. Our findings indicated that patients with LRP1B mutations exhibited higher expression levels of CD274, increased abundance of tumor‐infiltrating lymphocytes, elevated TMB, and improved survival outcomes. These results suggest that LRP1B mutations exert a positive influence on immunotherapy.

Subsequently, we further investigated the underlying mechanisms by which LRP1B affects immunotherapy. In our research, we discovered that LRP1B had the ability to control tumor cell susceptibility to ferroptosis induced by erastin. Specifically, LRP1B impacted the survival of tumor cells by modulating the phosphorylation level of the transcription factor STAT3, and consequently regulating the expression of SLC7A11.

Ferroptosis refers to a type of programmed cell death that is dependent on the presence of iron within cells. Iron and hydrogen peroxide in cells produce reactive oxygen species (ROS) through the Fenton reaction, which in turn triggers lipid peroxidation [33]. Maintaining intracellular redox balance is a crucial function of glutathione (GSH), which serves as a significant antioxidant in cells [34]. SLC7A11, a transporter of cystine, is essential for the synthesis of GSH [35]. Inhibition of SLC7A11 can trigger ferroptosis, while activation of SLC7A11 can prevent it. Our observation indicated a close correlation between the expression of SLC7A11 and LRP1B. Reducing LRP1B expression resulted in decreased SLC7A11 expression, as well as elevated levels of Lipid ROS and MDA induced by erastin in tumor cells. Overexpression of LRP1B increased the expression of SLC7A11 and inhibited the increase of lipid ROS and MDA induced by Erastin in tumor cells. When we overexpressed SLC7A11 in LRP1B knockdown cell lines, we rescued tumor cells from erastin‐induced ferroptosis, whereas knocking down SLC7A11 in LRP1B knockdown cell lines restored the sensitivity of tumor cells to erastin‐induced ferroptosis. In conclusion, our findings suggested that LRP1B modulated the susceptibility of LUAD cells to ferroptosis induced by erastin through the SLC7A11 axis.

The phosphorylation level of the transcription factor STAT3 is critical for ferroptosis. Some studies have shown that increasing the phosphorylation level of STAT3 can reduce ferroptosis caused by knocking down SLC7A11 [37], while LRP1B affects the phosphorylation of STAT3 [21]. In our study, we demonstrated that knockdown of LRP1B can inhibit STAT3 phosphorylation, while overexpression of LRP1B can promote STAT3 phosphorylation. Further double luciferase reporter gene experiments showed that increasing the STAT3 phosphorylation level could enhance the SLC7A11 promoter. Therefore, we believe that LRP1B affects the transcription of SLC7A11 by regulating the phosphorylation level of STAT3.

To summarize, our findings proposed that LRP1B mutation had the potential to serve as a biomarker for immunotherapy. Furthermore, our demonstration has revealed the regulatory role of LRP1B on ferroptosis through the STAT3‐SLC7A11 axis. Our findings offer valuable insights into the molecular mechanisms that govern LRP1B's function in controlling tumor cell death, and further support the possibility of utilizing LRP1B mutation as a biomarker for immunotherapy.

## Author Contributions

**Zi‐Hao Ke:** conceptualization (equal), formal analysis (equal), methodology (equal), validation (equal), writing – original draft (equal). **Ying Chen:** conceptualization (equal), methodology (equal), writing – review and editing (equal). **Tao Yu:** methodology (equal), supervision (equal), writing – original draft (equal), writing – review and editing (equal). **Qi Zhang:** data curation (equal), formal analysis (equal), validation (equal). **Yan Xiang:** data curation (equal), resources (equal), writing – review and editing (equal). **Kai‐Hua Lu:** conceptualization (equal), funding acquisition (equal), investigation (equal), supervision (equal).

## Ethics Statement

The use of patient data was approved by the institutional review boards of Jiangsu Province Hospital (No. 2022‐SR‐261). Animal experiments involved in this study were approved by the Committee on the Ethics of Animal Experiments of the Nanjing Medical University (No. IACUC‐2111042).

## Consent

The authors have nothing to report.

## Conflicts of Interest

The authors declare no conflicts of interest.

## Supporting information


**Figure S1.** In LUSC, the LRP1B mutation cannot serve as a biomarker for immunotherapy. (A) The expression of CD274 (PD‐L1) LUSC by LRP1B mutation status. (b) TMB in LUSC stratified by LRP1B mutation status. (C) Relative abundance of tumor‐infiltrating leukocytes in LRP1B mutant vs. LRP1B wild‐type samples in LUSC and significantly different tumor‐infiltrating leukocytes. The data were presented as mean ± SD (*** *p* < 0.001). (D) Kaplan–Meier survival analysis stratified by LRP1B mutation in LUSC (*n* = 21).
**Figure S2.** The construction of LRP1B knockdown cell and the construction of LRP1B overexpression cell. (A) The expression levels of LRP1B mRNA in various cell lines. (B and C) The detection of LRP1B overexpression and knockdown efficiency by qRT‐PCR and WB. The data were presented as mean ± SD (***p < 0.001; **p < 0.01; *p < 0.05).

## Data Availability

Data are available on reasonable request.
