# Supplementary material for: LRP1B Suppresses Immunotherapy Efficacy in Lung Adenocarcinoma by Preventing Ferroptosis
Source: Cancer Med. 2024 Dec 11;13(23):e70486. doi: 10.1002/cam4.70486 (PMC11632276; doi:10.1002/cam4.70486)
Supplement: Supplementary file 1 — Figure S1. In LUSC, the LRP1B mutation cannot serve as a biomarker for immunotherapy. (A) The expression of CD274 (PD‐L1) LUSC by LRP1B mutation status. (b) TMB in LUSC stratified by LRP1B mutation status. (C) Relative abundance of tumor‐infiltrating leukocytes in LRP1B mutant vs. LRP1B wild‐type samples in LUSC and significantly different tumor‐infiltrating leukocytes. The data were presented as mean ± SD (*** p < 0.001). (D) Kaplan–Meier survival analysis stratified by LRP1B mutation in LUSC (n = 21). Figure S2. The construction of LRP1B knockdown cell and the construction of LRP1B overexpression cell. (A) The expression levels of LRP1B mRNA in various cell lines. (B and C) The detection of LRP1B overexpression and knockdown efficiency by qRT‐PCR and WB. The data were presented as mean ± SD (***p < 0.001; **p < 0.01; *p < 0.05). [file CAM4-13-e70486-s001.docx]

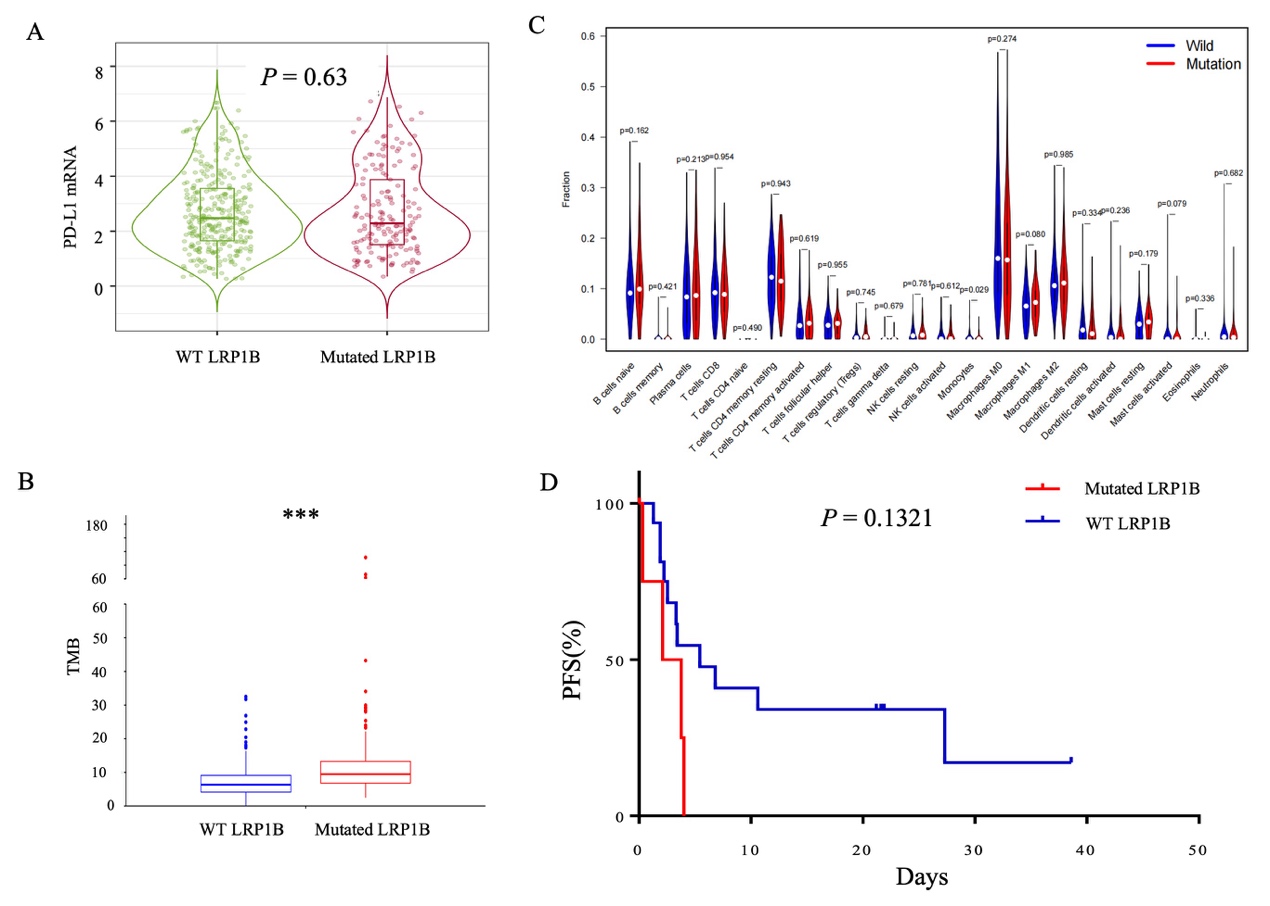


**Supplementary Figure 1. In LUSC, the LRP1B mutation cannot serve as a biomarker for immunotherapy. A.** The expression of CD274 (PD-L1) LUSC by LRP1B mutation status. **B.** TMB in LUSC stratified by LRP1B mutation status. **C.** Relative abundance of tumor infiltrating leukocytes in LRP1B mutant vs. LRP1B wild-type samples in LUSC and significantly different tumor-infiltrating leukocytes. The Data were presented as Mean ± SD (*** *P* < 0.001; ** *P* < 0.01; * *P* < 0.05). **D.** Kaplan-Meier survival analysis stratified by LRP1B mutation in LUSC (n = 21).


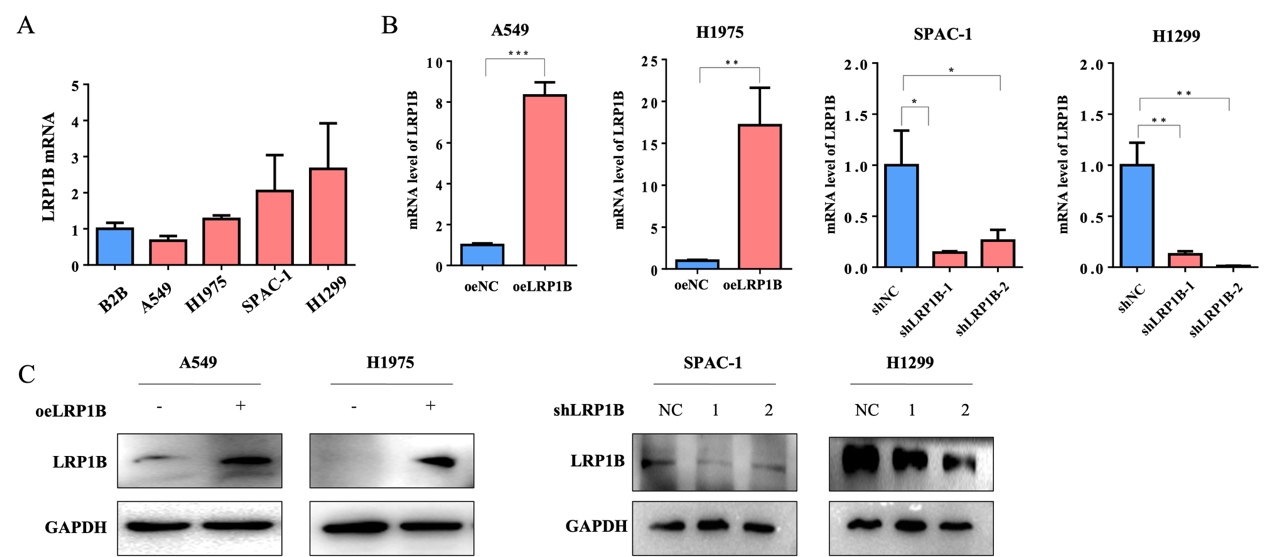


**Supplementary Figure 2. The construction of LRP1B knockdown cell and the construction of LRP1B overexpression cell. A.** The expression levels of LRP1B mRNA in various cell lines. **B** and **C.** The detection of LRP1B overexpression and knockdown efficiency by qRT-PCR and WB. The Data were presented as Mean ± SD (*** *P* < 0.001; ** *P* < 0.01; * *P* < 0.05).
